# Small cell lung carcinoma metastasis to palatine tonsils

**DOI:** 10.5935/1808-8694.20130117

**Published:** 2015-10-08

**Authors:** Helena Hotz Arroyo, Jefferson Takehara, Allex Itar Ogawa, Ronaldo Frizzarini, Rui Imamura, Henrique Moura de Paula

**Affiliations:** a MD, (Fellowship, Facial Plastic Surgery, Otorhinolaryngology Department, School of Medicine of the University of São Paulo, Brazil).; b MD (Preceptor, Otorhinolaryngology Program, School of Medicine of the University of São Paulo, Brazil).; c MD (Assistant Physician, Otorhinolaryngology-Oncology Program, São Paulo State Cancer Institute, Brazil).; d MD (Assistant Physician, Otorhinolaryngology Program, School of Medicine of the University of São Paulo, Brazil).; e MD, Pathologist (Preceptor, Pathology Division, School of Medicine of the University of São Paulo, Brazil). School of Medicine of the University of São Paulo, Brazil.

**Keywords:** lymphatic metastasis, oropharynx, palatine tonsil, small cell lung carcinoma, tonsillar neoplasms

## INTRODUCTION

Small-cell lung carcinomas (SCLC) are aggressive tumors^1^ which rarely metastasize to the palatine tonsils^2^, ^3^. Dissemination of disease to the tonsils seems to occur after antegrade movements of tumor cells through the neck lymphatic system^1^. The reasons that led the authors to report this case were the rarity of the occurrence in itself and the importance of carrying out thorough patient physical examination.

## CASE REPORT

A 64-year-old male individual came to our service complaining of dysphagia and odynophagia evolving for six months and a lesion in his left oropharynx. He had had dysphonia for two months and mild dyspnea while performing physical effort. The subject claimed antibiotics failed to improve his condition. He had been a smoker for 50 years.

**Figure 1:**
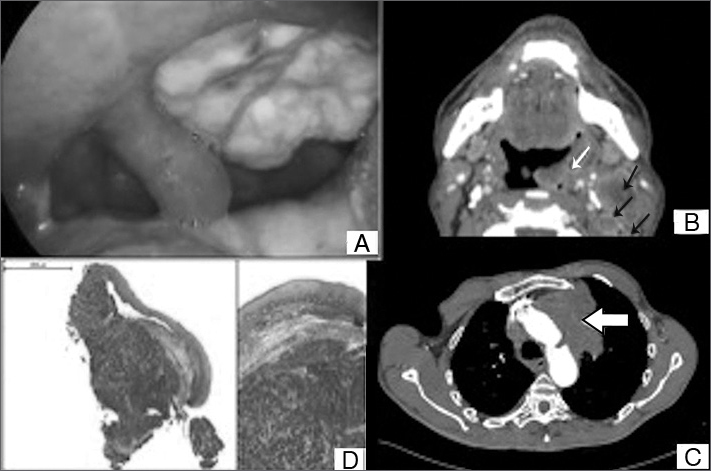
A: macroscopic view of a tumor in the left tonsillar bed; B: axial view of a neck CT scan (white arrow: tumor in the oropharynx; black arrows: lymph nodes); C: axial view of a chest CT scan (white arrow: tumor in the left upper lobe); D: squamous mucosa invaded by small-cell lung carcinoma characterized by mid-size trabecular tumor cells with round and oval nuclei and scarce cytoplasm; original magnification and 35x original magnification.

Physical examination revealed an ulcerated lesion of approximately four centimeters covered with fibrin in the left tonsillar bed (Figure 1A). Laryngoscopy showed he had left vocal fold paresis. Neck palpation showed coalescent nodes on levels II and III on the left side.

Neck CT scans revealed a tumor with heterogeneous contrast uptake infiltrating the left oropharynx and extending into the soft palate, associated with clustering nodes on levels IIA/IIB, with liquefied areas and signs of capsular leakage; other similar but smaller nodes were seen on levels III, IV, V, and VI (Figure 1B).

Chest CT scans showed lung emphysema and a tumor in the left upper lobe suggestive of lung carcinoma, in addition to enlarged mediastinal and bilateral supraclavicular lymph nodes (Figure 1C).

Pathology tests indicated the tonsillar tumor was a poorly differentiated carcinoma invading the squamous mucosa (Figure 1D). Immunohistochemistry (IHC) results were consistent with SCLC (CD45-negative, CD56-positive, cytokeratin 35BH11-positive, cytokeratin 5-positive, protein p63-negative, TTF1-negative, cromogranine-negative, synaptophysin-negative, ki67-positive).

Endobronchial biopsy indicated the patient had SCLC, as confirmed by IHC.

The patient died before the beginning of radiation and chemotherapy.

## DISCUSSION

Small-cell lung carcinoma is a relatively common type of lung carcinoma, accounting for 15-25% of the cases^1^, ^2^, ^3^. It is considered to be an aggressive tumor, as it involves a significant portion of the lung. Metastasis occurs early on and involves the liver, abdominal lymph nodes, bones, the brain, the adrenal gland, the skin, the kidneys, and the pancreas. Palatine tonsillar involvement is rare^1^, ^4^.

Palatine tonsillar metastasis originating from other primary tumors account for a small portion of tonsillar tumors (0.8%), and has been more frequently associated with breast, lung, kidney, testicular, skin, and rectal tumors^1^, ^5^, ^6^. Such pattern of disease spread may stem from antegrade movements of tumor cells through neck lymph nodes, the thoracic duct, and neck veins into the tonsils^1^.

A review covering 76 cases of tonsillar metastasis showed that ten of the 12 patients with lung cancer in the series had SCLC. In ten of the 12 cases there was evidence of metastasis in other tissues^5^.

Yaren et al.^2^ and Hisa et al.^3^ reported one case each of tonsillar metastasis by SCLC after treatment with chemoradiotherapy. Mastronikolis et al.^6^ reported cases of tonsillar tumors with metastasis to the liver, spleen, and ribs. Seddon et al.^1^ reported a case of tonsillar metastasis in a patient with SCLC and exuberant pulmonary symptoms.

Unlike other cases, our patient's ENT complaints were more marked than the pulmonary symptoms as a result of the tonsillar metastasis, which accounted for the disease's early manifestations.

Despite the poor prognosis usually seen for subjects with SCLC, late diagnosis may impact the chances of managing the disease. In the described case, the patient had been treated with antibiotics for having signs of disease consistent with tonsillitis. Accurate diagnosis was delayed because the patient was subject to inadequate interviews, lack of follow-up, and disregard for the possibility of his disease being cancer.

## CLOSING REMARKS

Given the simple nature of oropharynx examination and how often tumors in this area produce symptoms, careful oral examination must be performed even in patient with tumors in other sites. Tumors must be considered in patients with ulcerated lesions refractory to clinical management to expedite diagnosis and medical intervention.
